# Gastric volvulus with perforation 1 year after total pancreatectomy: a case report

**DOI:** 10.1186/s40792-020-00840-x

**Published:** 2020-04-17

**Authors:** Yusuke Takahashi, Hitoshi Seki

**Affiliations:** Department of Hepatobiliary Pancreatic Surgery, Nagano Municipal Hospital, 1333-1 Tomitake, Nagano City, Nagano, 381-8551 Japan

**Keywords:** Pancreatic intraductal neoplasms, Pancreatectomy, Stomach volvulus

## Abstract

**Background:**

Because of its rare indication and relatively simple reconstruction procedure (only choledochojejunostomy and gastrojejunostomy) compared to those for pancreatoduodenectomy, the technical tips and pitfalls of total pancreatectomy are rarely discussed. Herein, we discuss a rare case of gastric volvulus 1 year after total pancreatectomy and provide advice to prevent such cases.

**Case presentation:**

A 66-year-old woman underwent total pancreatectomy with splenectomy for mixed-type intraductal papillary mucinous neoplasm of the pancreas. Choledochojejunostomy (retro-colic route) and gastrojejunostomy (ante-colic route, Billroth II method) were performed for reconstruction. The final diagnosis was mixed-type intraductal papillary mucinous adenoma of the pancreas without malignant neoplasm. She had no clinical symptoms, such as abdominal pain and fever, during postoperative follow-up. However, at 1 year postoperatively, she complained of abdominal pain. Contrast-enhanced abdominal computed tomography showed volvulus and perforation of the stomach. Emergent surgery was performed. The stomach fornix was located on the right side and was partly perforated. We resected the perforation site with a linear cutter® (New Type Linear Cutter, Ethicon, USA) and released the gastric volvulus. Moreover, we fixed the stomach to the left abdominal wall using non-absorbable thread. The cause of the perforation was clinically and pathologically unclear. Her serum albumin and cholinesterase levels temporarily decreased postoperatively, but gradually increased. A recurrence of volvulus-related symptoms has not been observed.

**Conclusions:**

After total pancreatectomy with splenectomy, although the stomach is connected with the jejunum, it is typically fixed only by the pedicle of the left gastric artery and vein. In the present case, this anatomical change may have been a cause of the gastric volvulus. Thus, it might be better to fix the remnant stomach in total pancreatectomy with splenectomy.

## Background

Total pancreatectomy (TP) is rarely performed, but is sometimes indicated for specific widespread pancreatic carcinomas, main-duct (MD) or mixed-type (MD and branch duct (BD)) intraductal papillary mucinous neoplasms (IPMNs) of the pancreas, and chronic pancreatitis [1–3]. Because of its rare indication and relatively simple reconstruction procedure (choledochojejunostomy and gastrojejunostomy only) compared to that for pancreatoduodenectomy, the technical tips and pitfalls of TP are rarely discussed.

We experienced a patient with gastric volvulus 1 year after TP. A similar case has not been previously reported, although a rare case report of gastric volvulus following sleeve gastrectomy is reported [4]. Herein, we discuss this rare case and tips to prevent gastric volvulus after TP. The patient provided written consent to have her data published.

## Case presentation

**Fig. 1:**
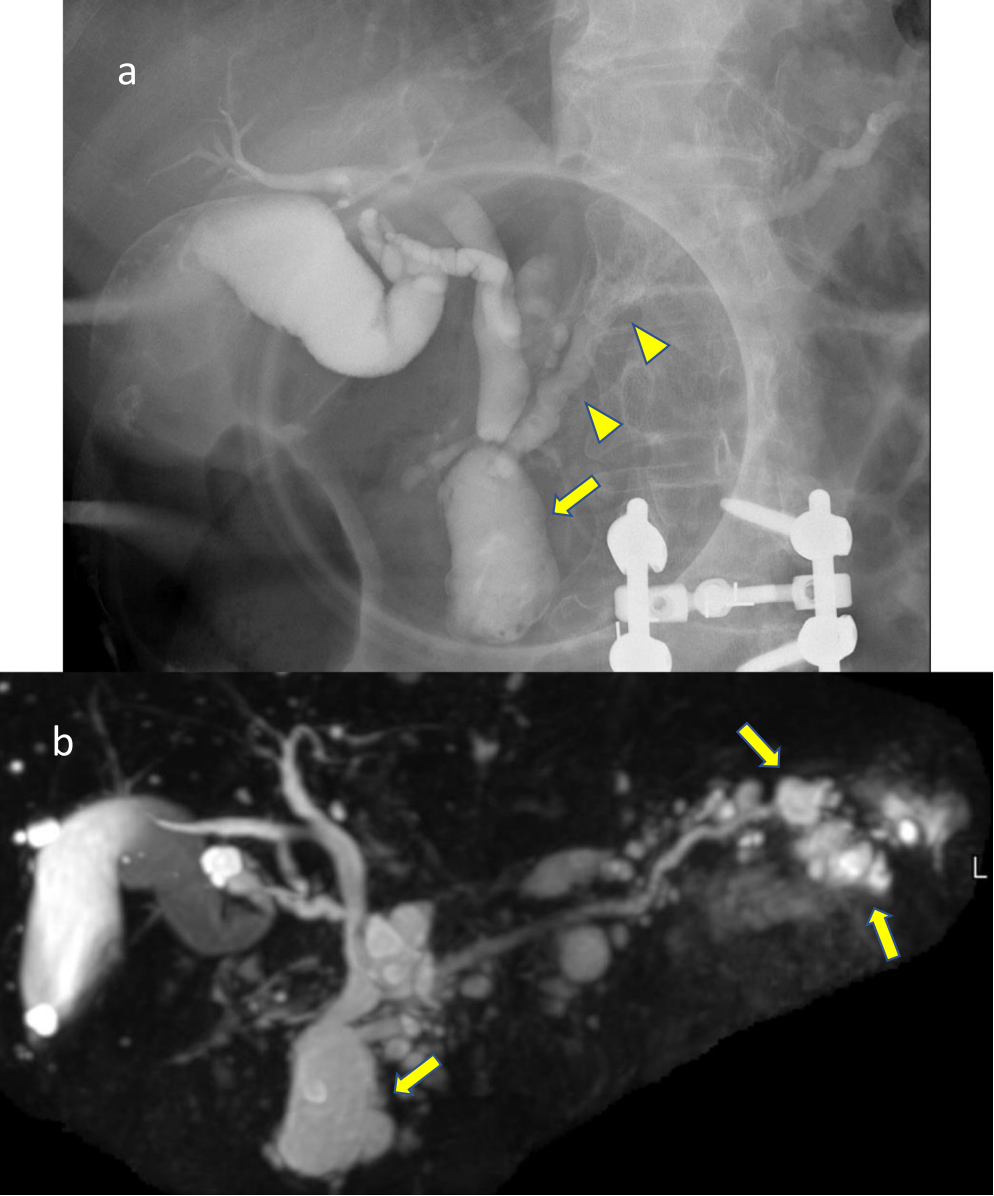
Cholangiopancreatographic images. **a** Endoscopic retrograde cholangiopancreatographic image. **b** Magnetic resonance cholangiopancreatographic image. Arrows: branch duct intraductal papillary mucinous neoplasm (IPMN). Arrowheads: main duct IPMN

A 66-year-old woman had a pancreatic cyst revealed by abdominal echography during a health examination. Endoscopic retrograde cholangiopancreatography (ERCP) and endoscopic ultrasonography showed a dilated diffuse main pancreatic duct (MPD) (diameter, 6 mm) and a BD-IPMN in the pancreatic head (size, 38 mm) without mural nodules (Fig. 1a). Magnetic resonance cholangiopancreatography (MRCP) showed a BD-IPMN in the pancreatic tail, in addition to the findings of ERCP (Fig. 1b). Mixed-type IPMN was diagnosed, with worrisome features of BD-IPMN in the pancreatic head. Contrast-enhanced computed tomography (CT) showed findings similar to those of ERCP and MRCP, with no significant lymph node swelling. Obstructive jaundice was not observed, and her serum carcinoembryonic antigen and carbohydrate antigen (CA) 19-9 levels were within normal ranges.

We planned subtotal stomach-preserving pancreatoduodenectomy (SSPPD) for BD-IPMN with worrisome features of the pancreatic head; however, she desired TP because of the potential risks of intraductal papillary mucinous carcinoma (IPMC) and pancreatic ductal adenocarcinoma (PDAC) in the remnant pancreas. Therefore, we performed TP and splenectomy with lymphadenectomy for mixed-type IPMN. The stomach was almost preserved, as in SSPPD. Choledochojejunostomy (retro-colic route) and gastrojejunostomy (ante-colic route, Billroth II) without Braun anastomosis were performed for reconstruction. Postoperative complications, such as cholangitis and delayed gastric empty, were not observed. Although she was not diabetic preoperatively, her blood sugar level was strictly controlled with continuous subcutaneous insulin infusion by an expert physician. The pathological diagnosis was mixed-type intraductal papillary mucinous adenoma of the pancreas.

**Fig. 2:**
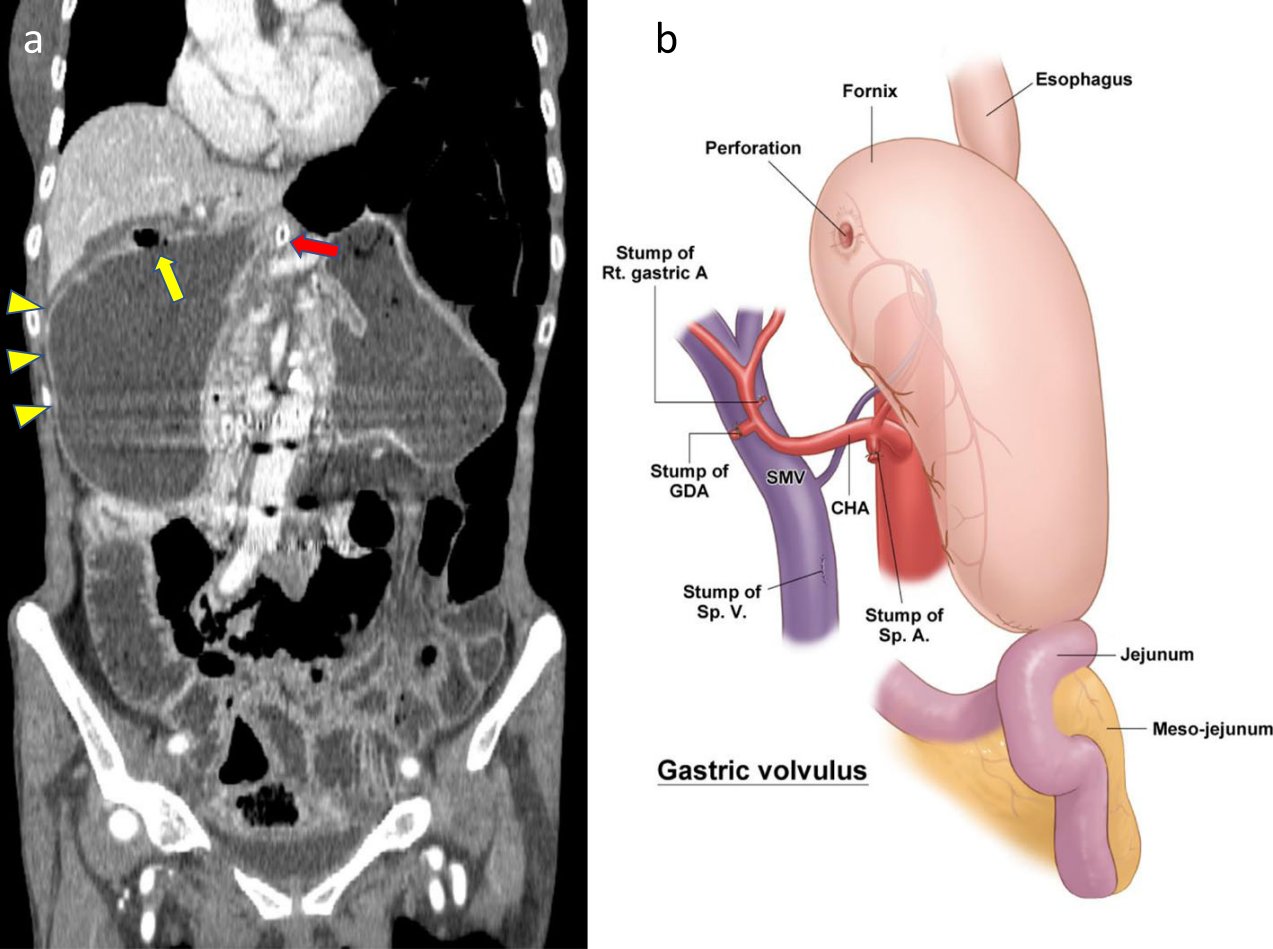
CT image and schema. **a** An organo-axial gastric volvulus and free air (yellow arrow) can be observed (coronal view). **b** Schema of gastric volvulus with perforation. Red arrows: naso-gastric tube, arrowheads: fornix of the stomach. rt. gastric A., right gastric artery; GDA, gastroduodenal artery; SMV, supramesenteric vein; CHA, common hepatic artery; Sp. A., splenic artery; Sp. V., splenic vein

**Fig. 3:**
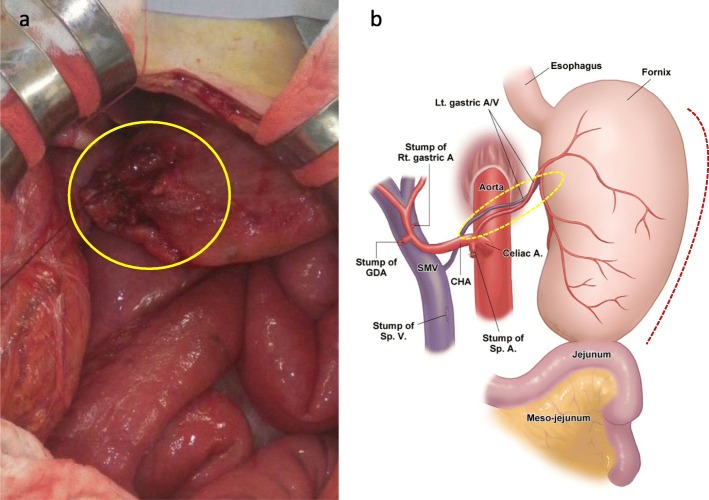
Intraoperative findings and schema following gastric volvulus release. **a** The yellow circle indicates the fornix of the stomach and perforated lesion. **b** The remnant stomach after total pancreatectomy with splenectomy is fixed only by the pedicle of the left gastric artery and vein (dotted circle). The greater curvature (dotted line) was fixed to the left abdominal wall. Celiac A., celiac artery; Lt. gastric A/V, left gastric artery and vein

Periodical follow-up (analysis of tumor marker levels, including carcinoembryonic antigen and CA19-9, every 3 months and contrast-enhanced CT every 6 months) was conducted in the outpatient clinic at our hospital. One year after surgery, she experienced abdominal pain. Contrast-enhanced CT showed an organo-axial volvulus of the stomach and free air (Fig. 2a, b). We performed emergent open surgery. Intraoperative findings were similar to the preoperative CT findings; an organo-axial volvulus of the stomach was observed, and part of the fornix, which was located on the right side, was perforated (Fig. 3a). We resected the perforated stomach with a linear cutter® (New Type Linear Cutter, Ethicon, USA) and released the volvulus. Moreover, the stomach (the greater curvature) was fixed to the left abdominal wall to prevent recurrence of gastric volvulus, using non-absorbable thread (intermittent suture, total 6 needles) (Fig. 3b). The postoperative course was uneventful, and she was discharged on postoperative day 13. Although her nutrition status temporarily decreased, it has gradually improved. One year after emergent surgery, recurrence of gastric volvulus has not been observed.

## Discussion

The treatment strategy for IPMN differs according to the various findings, including clinical data, such as an increased tumor marker (CA19-9) level and induced pancreatitis; cystic lesion properties, such as size, mural nodule presence, and wall thickness; and MPD diameter [2]. The surgical indications of MD-IPMN include MPD diameter > 10 mm, jaundice, and mural nodules. TP should be carefully selected, avoiding overtreatment [5]. In the present case, the patient was not jaundiced, the MPD diameter was only 6 mm, and mural nodules were absent. Thus, our initial surgical plan of SSPPD for BD-IPMN with worrisome features of the pancreatic head was considered to be valid.

However, the incidence of PDAC or IPMC in the remnant pancreas is an important issue; the 10-year cumulative incidences are reported as 8.8% and 38%, respectively [6, 7]. Recently, the long-term risk (15 years) of malignancy in BD-IPMN has been reported as 15% in Japan [8]. Thus, we informed the patient about our treatment strategy and future risk of PDAC or IPMC in the remnant pancreas. She was apprehensive of malignancy in the remnant pancreas in case of SSPPD and desired TP. Given the lack of apparent findings of malignancy in the body and tail of the pancreas, spleen-preserving TP was considered as a viable option for this case. However, this is, to our knowledge, the first case report of gastric volvulus after TP with splenectomy.

After TP with splenectomy, the stomach was anatomically fixed only by the pedicle of the left gastric artery and vein, despite gastrojejunostomy reconstruction. This procedure may have a potential risk of gastric volvulus. If the spleen is preserved, the risk of volvulus may be reduced because the remnant stomach is fixed by pedicles of the left gastric vessels and gastrosplenic ligament.

The reason for the accompanying perforation is unclear. In the present case, the subtotal stomach was preserved, and the left gastric artery, which feeds from the lesser curvature side, was main feeding artery. The distal side of the stomach might also be fed by the jejunal mesentery. The proximal greater curvature, including the fornix, may have lower blood flow than that of the other side, consequently leading to the perforation of a stressed and stretched fornix. Therefore, we resected the perforation lesion, instead of repairing with suture. Moreover, to prevent a recurrence of organo-axial volvulus of the stomach, we sutured the greater curvature to the left abdominal wall as gastropexy, using non-absorbable thread. This appears to be a favorable strategy, as no recurrence of gastric volvulus has been observed for 1 year after the surgery.

## Conclusions

In summary, the remnant stomach after TP with splenectomy may pose a risk of gastric volvulus and it might be better to fix this using non-absorbable thread. Spleen-preserving TP, in which the remnant stomach is fixed by both pedicles of left gastric vessels and gastrosplenic ligaments, should be considered in cases without high-risk stigmata or worrisome features in an IPMN of the pancreatic body and tail.

## Data Availability

The dataset supporting the conclusions of this article is available in the manuscript.

## Abbreviations

BD: Branch duct
CA: Carbohydrate antigen
CT: Computed tomography
ERCP: Endoscopic retrograde cholangiopancreatography
IPMC: Intraductal papillary mucinous carcinoma
IPMN: Intraductal papillary mucinous neoplasm
MD: Main duct
MPD: Main pancreatic duct
MRCP: Magnetic resonance cholangiopancreatography
PDAC: Pancreatic ductal adenocarcinoma
SSPPD: Subtotal stomach preserving pancreatoduodenectomy
TP: Total pancreatectomy
